# Adaptation and Psychometric Validation of the Greek Version of the Sensory Profile 2 Short Form

**DOI:** 10.3390/children13030315

**Published:** 2026-02-24

**Authors:** Panagiotis Pipelias, Christina Kanaka-Gantenbein, Katerina Papanikolaou, Xanthi Tigani, Maria Michou, Panagiota Pervanidou

**Affiliations:** 1 Postgraduate Program “The Science of Stress and Health Promotion”, School of Medicine, National and Kapodistrian University of Athens, 11527 Athens, Greece; ckanaka@med.uoa.gr (C.K.-G.); xanthitig@med.uoa.gr (X.T.); mariamixou@hotmail.com (M.M.); ppervanid@med.uoa.gr (P.P.); 2 Unit of Developmental and Behavioral Pediatrics, First Department of Pediatrics, School of Medicine, National and Kapodistrian University of Athens, “Aghia Sophia” Children’s Hospital, 11527 Athens, Greece; 3 First Department of Pediatrics, School of Medicine, National and Kapodistrian University of Athens, “Aghia Sophia” Children’s Hospital, 11527 Athens, Greece; 4 Department of Child and Adolescent Psychiatry, School of Medicine, National and Kapodistrian University of Athens, “Aghia Sophia” Children’s Hospital, 11527 Athens, Greece; katpapan@med.uoa.gr; 5 Department of Public and Community Health, School of Public Health, University of West Attica, 11521 Athens, Greece; 6 Human Ecology Laboratory, Department of Home Economics and Ecology, Harokopio University, 17676 Kallithea, Greece

**Keywords:** sensory, sensory profile, sensory processing, neurodevelopmental disorders, autism, ASD, ADHD, psychometric validation

## Abstract

**Highlights:**

**What are the main findings?**

- The Greek version of the Sensory Profile 2 Short Form (SSP2) demonstrated strong psychometric performance and excellent internal consistency in a nationwide sample of children and adolescents aged 3.0–14.11 years.
- Exploratory factor analysis identified a clear multidimensional structure, explaining 63.6% of the total variance.

**What are the implications of the main findings?**

- The Greek version of the SSP2 represents a reliable parent-report instrument for the assessment of sensory processing patterns in pediatric populations.
- Its availability facilitates standardized sensory assessment and supports both clinical practice and research in Greek-speaking settings.

**Abstract:**

**Background/Objectives**: Sensory processing constitutes a fundamental neurobiological mechanism that allows individuals to perceive, interpret, and respond adaptively to sensory input. Atypical patterns of sensory processing are frequently observed in neurodevelopmental disorders such as Autism Spectrum Disorder (ASD) and Attention-Deficit/Hyperactivity Disorder (ADHD). The purpose of this study was to evaluate the psychometric properties of the Greek Sensory Profile 2 Short Form following its linguistic and cross-cultural adaptation. **Methods**: An on-site parent-report survey was conducted among children and adolescents aged 3.0 to 14.11 years across Greece. The study included the Sensory Profile 2 Short Form (SSP2), the Child Behavior Checklist (CBCL), and the Social Communication Questionnaire (SCQ). A total of 350 parents participated in the study. **Results**: The present study demonstrated that the Greek version of the SSP2 possesses good psychometric properties. The results of the Exploratory Factor Analysis (EFA) of the 34 items explained 63.6% of the total variance. Internal consistency for both primary scales—Sensory (α = 0.93) and Behavioral (α = 0.96)—was satisfactory, as were the subscales Seeking (α = 0.88), Avoiding (α = 0.94), Sensitivity (α = 0.94), and Registration (α = 0.91). Significant correlations with the CBCL and SCQ supported convergent validity. The findings also highlighted associations with sociodemographic variables, providing insights into population-specific variations in sensory processing. **Conclusions**: The Greek version of the SSP2 demonstrates strong psychometric properties and is suitable for assessing sensory processing patterns in children and adolescents. Its use enables culturally appropriate screening for atypical sensory processing, informing both research and clinical practice and supporting early identification and intervention strategies.

## 1. Introduction

The development of the sensory system begins prior to birth, highlighting the fundamental role of biological maturation in early psychophysiological growth [1]. From the moment of birth, infants exhibit an innate tendency to respond to sensory stimuli, forming the basis for their interaction with the external environment [2]. Multisensory experiences are deeply embedded in everyday life and constitute a critical foundation for individuals’ active participation in diverse tasks and activities. Sensory registration refers to the initial detection and recording of sensory stimuli by specialized receptors within the body [3]. The nervous system receives sensory input through receptor structures located in peripheral organs and tissues. These receptors function by transducing external stimuli into neural or electrical signals, which are subsequently transmitted to the brain through sensory nerves and pathways [4]. Sensory processing is a neurobiological process that encompasses the transmission, interpretation, and ultimately the adaptive response to sensory inputs [5]. This process constitutes a fundamental prerequisite for perception and for the individual’s ability to interact effectively with the surrounding environment. Depending on the nature of the stimulus, sensory information is processed within distinct cortical and subcortical regions of the brain [6], which function as an integrated network, guiding appropriate behavioral responses.

The types of sensory processing are principally conceptualized through Winnie Dunn’s (1997) [7] framework, which asserts that sensory processing patterns are determined by two principal dimensions: the neurological threshold and the behavioral response strategy, also referred to as mechanisms of self-regulation [8]. The neurological threshold refers to the intensity of sensory input for detection by the nervous system and the subsequent elicitation of a neurological response, ranging from high thresholds, which result in slower detection, to low thresholds, which facilitate rapid detection. The response strategy pertains to the manner in which individuals actively or passively engage with sensory stimuli. The dynamic interplay between these two dimensions gives rise to four distinct sensory processing patterns [9]: low registration (individuals with a high threshold who passively perceive stimuli and do not seek additional input), sensory sensitivity (individuals with a low threshold who passively detect stimuli but do not attempt to avoid them), sensory seeking (individuals with a high threshold who actively pursue intense sensory experiences), and sensory avoidance (individuals with a low threshold who actively attempt to avoid sensory stimuli) [7].

The theory of Sensory Integration, as developed by A. Jean Ayres, conceptualizes sensory integration as “the neurological process that organizes sensory input from the body and the environment and enables effective use of the body within the environment” [10]. A holistic organization and regulation of sensory information is essential for generating adaptive responses in daily life, yielding outcomes that manifest across multiple domains, including motor [11], cognitive [12], perceptual, behavioral, learning [13], and emotional functions [14]. Sensory regulation constitutes a neurobiological process within the central nervous system that enables contextually appropriate and functionally adaptive responses to sensory stimuli [15]. Accordingly, the behavioral repertoire of a child is profoundly shaped by their pattern of sensory processing [16]. Sensory registration, processing, and integration involve all human sensory systems, including the visual, auditory, olfactory, gustatory, tactile, vestibular, proprioceptive, and interoceptive systems.

In recent years, an increasing number of studies has examined atypical sensory processing, characterized by impairments in registration, interpretation, and integration of sensory stimuli [17]. Estimates suggest that sensory processing difficulties affect approximately 3–16% of the general population [18] and are substantially more prevalent among individuals with neurodevelopmental disorders (NDDs), with reported prevalence rates ranging from 20% to 95% depending on the methodological approach and sample characteristics [19]. Given that sensory processing difficulties are not confined solely to individuals with NDDs but are also observed in neurotypical individuals, several authors have proposed the designation of a distinct diagnostic category referred to as Sensory Processing Disorder (SPD) [20]. It is important to distinguish between sensory processing difficulties, which may occur in typically developing children and reflect normal variability in sensory responsiveness, and SPD, a proposed but controversial diagnostic category associated with clinically significant functional impairment. Consequently, the presence of atypical sensory responses does not necessarily indicate SPD. Several studies have demonstrated that children with SPD exhibit reduced participation in daily activities, including eating, sleeping, dressing, toileting, and personal hygiene, as well as in recreational pursuits, compared to their typically developing peers [21,22,23]. Furthermore, SPD has been positively associated with maladaptive behaviors, which may include aggression, irritability, hyperactivity, intense temper outbursts, and self-injurious actions [24].

Atypical sensory processing has been identified as a core feature of Autism Spectrum Disorder (ASD) since Kanner’s earliest clinical descriptions [25]. In the DSM-5, hyper- and hypo-reactivity to sensory input were incorporated as supplementary diagnostic criteria for the disorder [26]. Research on sensory processing in children with ASD indicates that up to 95% exhibit hyper- and/or hypo-sensitivity across multiple sensory modalities [27]. Several authors have identified distinct sensory processing patterns in individuals with ASD, which are directly associated with the clinical characteristics of the disorder [28]. According to Ayres [29], children with ASD not only demonstrate difficulties in accurately registering sensory stimuli but also encounter challenges in processing and organizing incoming sensory information. Consequently, atypical sensory registration, processing, and modulation may lead to dysfunctions in sensory integration. Moreover, a substantial body of evidence indicates that children with Attention-Deficit/Hyperactivity Disorder (ADHD) also differ in their sensory processing compared to neurotypical peers [30,31]. ADHD and SPD exhibit significant overlap in symptomatology, such as inattention, impulsivity, and hyperactivity, suggesting similar neurobiological and pathophysiological mechanisms. Studies indicate that between 40–84% of children with ADHD also meet the criteria for SPD, and vice versa [32]. Also, some researchers have proposed that atypical tactile sensory processing in early childhood may serve as a predictor of future attentional dysfunction, thereby contributing to the early identification of NDDs, particularly ADHD [33]. Additionally, several studies have documented associations between sensory processing and sociodemographic factors, including sex, marital status, and socioeconomic status. Among typically developing children, lower parental education level has been linked to sensory processing challenges [34,35]. Notably, despite the observed positive correlation between NDDs and SPD, children with similar sensory processing profiles but different NDDs may reveal divergent behavioral patterns [30]. Although atypical sensory processing is frequently observed in both NDDs and typically developing children, this highlights the need for a culturally adapted and reliable assessment tool for the Greek population.

The Sensory Profile, developed by Dunn, constitutes the most widely utilized instrument for evaluating sensory processing [36]. The Short Form of the Sensory Profile 2 was selected for this study due to its brevity, making it feasible for administration in large-scale community samples while maintaining robust psychometric properties. In addition, its design is suitable for screening purposes, allowing for the efficient identification of children who may exhibit atypical sensory processing patterns.

Based on theoretical considerations, we formulated a priori hypotheses. We expected the Greek version of the Sensory Profile 2 Short Form to reflect its two primary domains—Sensory and Behavioral—and to demonstrate convergent validity with behavioral and communicative questionnaires, as well as potential associations with sociodemographic factors. Specifically, the study aimed to examine (1) the reliability of the Greek version of the Sensory Profile 2 Short Form, including internal consistency; (2) the construct validity of its two primary domains—Sensory and Behavioral—and their associations with external measures; and (3) its criterion/concurrent validity through analyzing its relationships with sociodemographic variables and other instruments.

Despite the Sensory Profile being widely used internationally, there is currently no validated Greek version available. Previous validation studies in other languages have demonstrated the Short Form’s utility for assessing sensory processing but cross-cultural differences underscore the need for a locally adapted instrument. Given the absence of a Greek version, it is unclear whether existing norms are applicable to Greek children, creating a gap in both research and clinical practice. This study addresses this gap by providing a psychometric evaluation of the Greek version of the Sensory Profile 2 Short Form, ensuring culturally appropriate assessments and supporting early identification of atypical sensory processing patterns. In light of the central role of sensory processing in clinical practice, the present study sought to evaluate the psychometric properties of the Greek version of Sensory Profile 2 Short Form, following its linguistic and cross-cultural adaptation, assessing its reliability and validity. Validating the Greek version in a general population sample provides clinically meaningful insights, enabling practitioners to screen efficiently for atypical sensory processing patterns, interpret scores in both typical and clinical populations, and inform early identification and intervention strategies.

## 2. Materials and Methods

### 2.1. Translation Procedure

The translation of Sensory Profile 2 Short Form was conducted in accordance with the World Health Organization (WHO) guidelines for the translation and adaptation of instruments [37]. The initial forward translation (from English to Greek) was carried out by two bilingual occupational therapists with experience in administering the instrument and training in Sensory Integration. A third bilingual therapist resolved any discrepancies between the translated and original versions. Subsequently, the Greek version underwent backward translation by two bilingual professionals with no prior familiarity with the instrument (phase 2). In addition, a cognitive debriefing process, involving pre-testing of the translated questionnaire with a small subset of the sample, was conducted to ensure clarity and cultural appropriateness.

### 2.2. Participants and Procedures

The study was conducted on a sample of 350 children and adolescents aged 3.0 to 14.11 years from across Greece (Attica, mainland Greece, and islands). The sample size was determined to meet recommended criteria for factor analysis. Specifically, guidelines suggest a minimum of 5–10 participants per questionnaire item. With 34 items in the Sensory Profile 2 Short Form, a sample of 350 exceeds the recommended minimum, ensuring stable factor extraction and reliable estimation of psychometric properties.

The sample was qualitatively comparable to that of the original instrument, comprising children and adolescents from the general population. As the questionnaire was administered to parents of children/adolescents from the general population without exclusion criteria, it was anticipated that the sample would include individuals with NDDs at prevalence rates consistent with those observed in the general population. Participants were recruited using a convenience sampling strategy. Invitations to participate were distributed to parents via schools, pediatric clinics, and community centers across urban and rural areas of Greece. This approach aimed to capture a diverse sample reflecting the general population. While a formal assessment of sampling bias was not conducted, the demographic characteristics of the sample (age, sex, region, parental education, etc.) were compared with national census data to ensure reasonable representativeness.

### 2.3. Ethical Considerations

For the purposes of the study, permission to use the instrument was obtained from the publisher and copyright holder, Pearson’s Clinical Assessment Group, as well as from the author of the questionnaire, Professor Winnie Dunn. The study’s protocol was approved by the Ethics Committee of the Medical School of the National and Kapodistrian University of Athens, and was conducted in accordance with the Declaration of Helsinki (2013). Also, the study was approved by the Scientific and Ethics Committee of “Aghia Sofia” Children’s Hospital. For the completion of the printed questionnaires, volunteers who participated in the study signed a form of informed consent and confidentiality.

### 2.4. Measures

Participants were asked about their sex, age, nationality, marital status, place of residence, educational level, employment status, occupation, number of children, birth order, relationship to the child, as well as the sex and age of their child.

Sensory Profile 2 Short Form (SSP2): SSP2 is a caregiver-reported questionnaire consisting of 34 items, specifically designed to assess behavioral responses to sensory stimuli in children aged 3.0 to 14.11 years. The questionnaire classifies sensory processing patterns into four quadrants, based on the child’s neurological threshold to sensory input and corresponding self-regulatory strategies [38]. These quadrants include sensory seeking, sensory avoidance, sensory sensitivity, and low registration. Psychometric analyses indicate satisfactory internal consistency for each quadrant, with Cronbach’s alpha values as follows: sensory seeking (α = 0.69; 7 items), sensory avoidance (α = 0.83; 9 items), sensory sensitivity (α = 0.75; 10 items), and low registration (α = 0.75; 8 items). Additionally, the reliability of the broader domains of sensory processing and behavioral responses is robust (α = 0.88 and α = 0.90, respectively). Caregivers rate children’s typical responses to stimuli and the frequency of observed behaviors using a 5-point Likert scale ranging from 1 (almost never = 10% or less) to 5 (almost always = 90% or more), with higher scores reflecting greater sensory processing difficulties. In accordance with the original SSP2 administration and scoring guidelines, responses marked as “Does not apply” are coded as 0; this coding is part of the original instrument format and was retained in the present study without modification. Separate scores are derived for each quadrant, which are subsequently classified into five interpretive categories: much less than other children, less than other children, similar to the majority of other children, more than other children, and much more than other children [39].

Child Behavior Checklist (CBCL): CBCL is a widely used parent-report instrument for assessing behavioral, emotional, and social problems in children [40]. It covers domains including competence, anxiety/depression, withdrawal, somatic complaints, social, thought, and attention problems, as well as delinquent and aggressive behaviors, internalizing and externalizing problems, and specific conditions such as ADHD, oppositional defiant disorder, obsessive–compulsive disorder, post-traumatic stress disorder, sluggish cognitive tempo, and pervasive developmental problems [41]. Two age-specific forms are available: the CBCL/1.5–5 years (100 items) and the CBCL/6–18 years (113 items), both rated on a 3-point Likert scale (0–2), with total scores derived from summed item responses. Data are processed through the ASEBA software, ADB Version 8 (Achenbach System of Empirically Based Assessment, Burlington, VT, USA), which generates T-scores based on Greek population norms. The CBCL has been translated and standardized in Greece by the Association for the Mental Health of Children and Adolescents [42], and its reliability and validity have been extensively documented, supporting its use in clinical, educational, and research contexts.

Social Communication Questionnaire (SCQ): SCQ is a 40-item parent-report screening tool designed to detect symptomatology associated with ASD [43]. It can be completed in less than 10 min and evaluates behavior over the previous three months, providing information relevant to daily functioning, treatment evaluation, and educational planning. The SCQ has been translated and standardized in the Greek population [44]. In the Greek version, items are scored dichotomously (Yes/No), with items 3–18 positively scored and items 2,9, and 19–40 reverse scored. Item 1 determines whether all items (2–40) or only items 8–40 are included in the final calculation, and the total score is derived by summing responses across four columns. SCQ is widely used in clinical practice and research as a reliable tool for assessing autism-related symptomatology in individuals with ASD or other NDDs and typical development.

### 2.5. Statistical Analysis

Descriptive characteristics of the participants were presented as frequencies (%) for categorical variables and as medians with interquartile ranges for quantitative variables. The Kolmogorov–Smirnov test was applied to assess normality. Construct validity was assessed via Exploratory Factor Analysis (EFA), with sample adequacy evaluated using the Kaiser–Meyer–Olkin (KMO) test and Bartlett’s test of sphericity. Although the SSP2 factor structure is well-established internationally, only EFA was conducted in the present study. This decision was based on the cross-cultural adaptation context: EFA allows for the exploration of the underlying factor structure within the Greek population and identifies potential cultural variations in item loadings, prior to any confirmatory testing. Internal consistency was examined using Cronbach’s alpha. Non-parametric tests and correlation coefficients were applied to identify statistically significant differences and associations among variables. Cut-off points were derived from the percentile distribution of the normative sample following the ±1 SD convention used in the original SSP2. All analyses were conducted using SPSS v.26 [45], with the significance level set at *p* < 0.05.

## 3. Results

In the present study, 350 parents participated by completing the questionnaires mentioned above, which assessed the sensory, behavioral, and communicative profiles of their children. Participants’ sociodemographic characteristics are presented in Table 1. In total, the median age of the parents was 39 years (IQR = 10.25), and the majority of participants were mothers (65.1%). Most children were boys (55.1%), with a median age of 6.11 years (IQR = 4.04). Most parents were married (85.4%), of Greek nationality (95.4%), had post-secondary or tertiary education (58.9%), and were employed (90.3%). On average, most families had two children (IQR = 1), and in the majority of households (89.4%), there were no more than three children. Finally, regarding the results of the SCQ, presented in the same table, the mean score was 5 (IQR = 6)

Exploratory Factor Analysis (EFA) was conducted to identify the underlying dimensions (factors) accounting for the correlations among the 34 items of the SSP2 questionnaire. The adequacy of the sample was assessed using the Kaiser–Meyer–Olkin (KMO) test, which yielded a KMO coefficient of 0.965, while Bartlett’s test of sphericity indicated that factor analysis was statistically significant (*p* < 0.001). The EFA revealed that two factors had eigenvalues greater than 1, explaining 63.6% of the total variance. All item loadings were greater than 0.531, demonstrating satisfactory factor saturation.

The EFA of the Greek version of the SSP2 supported a two-factor structure, corresponding to the Sensory and Behavioral domains of the instrument. These two higher-order factors reflect the overarching organization of the questionnaire, whereas the four quadrants—Sensory Seeking, Sensory Avoiding, Sensory Sensitivity, and Low Registration—represent specific patterns of sensory processing within these broader domains. This structure aligns with the theoretical framework of the SSP2, in which the quadrants provide detailed information regarding specific sensory response patterns, while the two primary domains summarize overall sensory and behavioral functioning.

To assess the internal consistency of the SSP2, Cronbach’s alpha coefficients were calculated, which indicate the extent to which the items reliably measure the same construct. Cronbach’s α values were computed for the two primary scales (Sensory and Behavioral) and the subscales (Seeking, Avoiding, Sensitivity, and Registration) of the SSP2 and are presented in Table 2. The results demonstrated good internal consistency, with alpha values for the two scales ranging from 0.93 to 0.96, and ranging from 0.88 to 0.94 for the four subscales. All items’ total correlations were greater than 0.551. Additionally, Table 2 presents the descriptive statistics (mean, median, range, minimum, and maximum) for the SSP2 scales and subscales.

Table 3 presents the significant differences between the two primary scales (Sensory and Behavioral) of the SSP2 and the sociodemographic characteristics of the participants. For the Sensory scale, statistically significant differences were observed between groups based on parent’s sex (*p* = 0.001), nationality (*p* = 0.005), educational level (*p* = 0.018), father’s occupation (*p* = 0.039), children’s sex (*p* = 0.003), and birth order (*p* = 0.005). Regarding the Behavioral scale, statistically significant differences were found in relation to parent’s sex (*p* = 0.001), marital status (*p* = 0.043), nationality (*p* = 0.002), educational level (*p* = 0.019), father’s occupation (*p* = 0.044), children’s sex (*p* = 0.001), and birth order (*p* = 0.002). More specifically,

- Parent’s sex: Fathers rated their children’s behaviors higher on both the Sensory and Behavioral scales compared to mothers.
- Marital status: Parents who were unmarried, separated, divorced, widowed, or in single-parent families or in a civil partnership reported higher scores on the Behavioral scale compared to married parents.
- Nationality: Parents of Albanian, American, or Cypriot nationality reported higher scores for their children on both the Sensory and Behavioral scales compared to parents of Greek nationality.
- Educational level: Children of parents with primary or secondary education scored higher on the Sensory scale compared to those whose parents had post-secondary, tertiary, or postgraduate/doctoral education. Similarly, children of parents with primary or secondary education scored higher on the Behavioral scale compared to those of parents with post-secondary or tertiary education.
- Father’s occupation: Children whose fathers were unemployed or retired scored higher on both the Sensory and Behavioral scales compared to children whose fathers were civil servants or self-employed.
- Children’s sex: Boys had higher scores on both the Sensory and Behavioral scales compared to girls.
- Birth order: Only children scored higher on both the Sensory and Behavioral scales compared to first- or second-born children.

**Table 3:** Association of sociodemographic characteristics with the SSP2 scales (*n* = 350).

Characteristics	Category	Sensory Scale	Behavioral Scale
Sex of Parent Median (IQR)	Male	22 (15)	33 (27)
	Female	17 (11)	26 (17)
	*p*-value	0.001	0.001
Marital Status Median (IQR)	Married	18 (12)	28 (19)
	Other	22 (22)	34 (33)
	*p*-value	0.055	0.043
Nationality Median (IQR)	Greek	19 (12)	28 (19.25)
	Other	28 (13.75)	40 (31.75)
	*p*-value	0.005	0.002
Place of residence Median (IQR)	Attica	18 (13.5)	29 (20)
	Mainland Greece	19.5 (11.25)	29.5 (20.5)
	Islands	21.5 (12.5)	27 (20)
	*p*-value	0.935	0.196
Educational Level Median (IQR)	Until Upper Secondary School	22 (23) *^	36 (44) *
	Post-Secondary/Tertiary	19 (12.25) *	28 (20) *
	MSc/PhD	18 (11) ^	29 (18)
	*p*-value	0.018	0.019
Work Status Median (IQR)	Employed	18.50 (12)	28.50 (19.75)
	Other	23 (13.25)	33 (18.75)
	*p*-value	0.085	0.211
Fathers’ Occupation Median (IQR)	Public Sector Employee	19 (10) ^	29 (21) ^
	Private Sector Employee	20 (15)	31 (23)
	Self-employed	18 (11) *	27 (15.5) *
	Other	38 (23.5) *^	62 (44) *^
	*p*-value	0.039	0.044
Mother’s Occupation Median (IQR)	Public Sector Employee	18.5 (11)	27.5 (15.75)
	Private Sector Employee	20 (14)	32 (25)
	Self-employed	17 (11)	27 (17.5)
	Other	23 (16.5)	31 (29.5)
	*p*-value	0.089	0.183
Sex of Child Median (IQR)	Male	21 (14)	33 (23)
	Female	17 (10)	26 (15)
	*p*-value	0.003	0.001
Birth Order Median (IQR)	Only Child	22 (18.25) *^	34 (25.25) *^
	1st	17 (11) *	26.5 (15.25) *
	2nd	18 (14.25) ^	28 (20.75) ^
	Other	18 (8.75)	26 (19)
	*p*-value	0.005	0.002
More Than 3 Children Median (IQR)	Yes	18 (10)	27 (19.5)
	No	19 (12.5)	30 (21)
	*p*-value	0.393	0.139

Note: The symbols (*, ^) indicate statistically significant pairwise differences between the specified groups at *p* < 0.05. Detailed group comparisons are provided within the table.

Table 4 presents the Spearman’s rho correlations between SSP2 scales and the study’s quantitative variables. A strong positive correlation was observed between the two primary SSP2 scales (Sensory and Behavioral, r = 0.872, *p* < 0.001). Moderate positive correlations were found between the Sensory scale and SCQ (r = 0.311, *p* < 0.001) and between the Behavioral scale and SCQ (r = 0.326, *p* < 0.001). Both SSP2 scales were negatively correlated with parent age, child age, and number of children. Specifically, the Sensory scale showed moderate negative correlations with parent age (r = −0.328, *p* < 0.001) and child age (r = −0.394, *p* < 0.001), as well as a weak negative correlation with the number of children (r = −0.165, *p* = 0.002). Similarly, the Behavioral scale was moderately negatively correlated with parent age (r = −0.340, *p* < 0.001) and child age (r = −0.4, *p* < 0.001), and weakly negatively correlated with the number of children (r = −0.178, *p* = 0.001).

Children classified within the clinical range of the CBCL scored significantly higher on both the Sensory and the Behavioral SSP2 scales compared to children in the non-clinical range. This indicates a clear relationship between atypical sensory processing and broader behavioral and emotional difficulties. Significant positive associations were observed across key CBCL domains, including social competence, school performance, internalizing (anxiety–depression, withdrawal) and externalizing (aggressive behavior, emotional reactivity) problems, attention problems, affective problems, post-traumatic stress symptoms, disruptive behaviors (oppositional defiant and conduct problems), and pervasive developmental problems. These findings suggest that higher Sensory and Behavioral scores on the SSP2 are strongly linked to a broad spectrum of behavioral and emotional difficulties, supporting the concurrent validity of the questionnaire in identifying children at risk.

Based on the standardized Greek version of the questionnaire, new cut-off points were established to ensure appropriate interpretation of SSP2 scores within the Greek population and for the purposes of the present study. These cut-off points were derived from the normative sample (*n* = 350) by examining the percentile distribution of scores. New cut-off points were established for each domain and subscale. Following the conventional SSP2 approach, the newly defined thresholds correspond approximately to ±1 standard deviation from the mean: scores below the 16th percentile are classified as “Less Than Others,” scores between the 16th and 84th percentiles are classified as “Just Like the Majority of Others,” and scores above the 84th percentile are classified as “More Than Others”. Table 5 presents the cut-off values resulting from the standardization of the SSP2 for the Greek population.

## 4. Discussion

This study aimed to adapt and validate the Sensory Profile 2 Short Form questionnaire for the Greek language and population. The psychometric properties of the Greek version of the SSP2 were examined in a sample of 350 children and adolescents aged 3.0 to 14.11 years from the general population. The SSP2 was developed to assess children’s sensory and behavioral responses to everyday stimuli, offering insights into the organization and responsiveness of the nervous system to sensory input [39]. Determining a child’s sensory processing profile—that is, the manner in which the child registers, interprets, and responds to sensory stimuli from the environment—can provide valuable information and substantially inform diagnostic assessment, developmental understanding, behavioral interpretation, individualized intervention planning, and daily functional outcomes [46]. In addition to the original English version, the questionnaire has been standardized in Poland [47] and Malaysia [48]. The instrument is distinguished by its high reliability and robust psychometric properties.

Similarly, the statistical analysis of the present Greek standardization demonstrated excellent internal consistency for both primary scales—Sensory (α = 0.93) and Behavioral (α = 0.96)—as well as for the subscales Seeking (α = 0.88), Avoiding (α = 0.94), Sensitivity (α = 0.94), and Registration (α = 0.91). Although the high Cronbach’s alpha values indicate excellent internal consistency, they may also reflect some degree of item redundancy. This is consistent with previous SSP2 validations and does not compromise the overall reliability of the instrument [39]. Additionally, Exploratory Factor Analysis (EFA) confirmed that the 34 items of the SSP2 load onto two factors, which together account for a substantial proportion of the total variance (63.6%). The two primary factors identified in the Greek SSP2—Sensory and Behavioral—correspond to broader domains encompassing the original four-quadrant model. Specifically, the four quadrants (Seeking, Avoiding, Sensitivity, Registration) are conceptually nested within these two higher-order domains. This approach aligns with prior cross-cultural adaptations of the SSP2, where higher-order aggregation into two primary domains has been reported. Importantly, this does not alter the theoretical relevance of the original four quadrants, which remain conceptually meaningful within the Sensory and Behavioral domains. This structural simplification maintains theoretical consistency with Dunn’s model while facilitating interpretability for research and screening purposes. Furthermore, a highly statistically significant positive correlation was observed between the two primary scales (Sensory and Behavioral) of the SSP2, with a correlation coefficient of r = 0.872 (*p* < 0.001), indicating substantial interrelatedness between sensory processing and behavioral responses. This substantial interrelatedness between the Sensory and Behavioral scales confirms that in this population, sensory processing and its behavioral manifestations are deeply integrated, supporting the use of these broader scales for efficient screening and intervention planning.

The results of the standardized Greek version of the questionnaire also revealed significant associations with sociodemographic factors, contributing to a more comprehensive understanding of children’s sensory profiles. Specifically, male sex, younger age, lower parental educational level, non-married family status, and absence of siblings were associated with increased sensory difficulties in children. The Discussion focuses on sociodemographic variables most relevant to sensory and behavioral outcomes, highlighting patterns with consistent clinical or theoretical relevance. It is important to note that these analyses are exploratory, and the observed associations should not be interpreted as causal. The large number of comparisons increases the risk of Type I error; thus, these findings provide preliminary insights and require confirmation in future studies. Sensory processing is a critical component of neurodevelopmental functioning and has been linked to key domains of daily behavior and participation [49]. While its relationship with NDDs, such as ASD, is well established [50,51,52,53], the influence of sociodemographic variables on sensory functioning has only recently begun to be systematically examined.

Population-based studies indicate that certain sociodemographic factors are associated with variations in sensory processing. For example, a large-scale study of 8.397 children aged approximately 8 years reported a prevalence of sensory processing difficulties of 8.3% in the general population and 53.6% among children with ASD [54]. Additionally, boys were more likely to exhibit atypical sensory processing regardless of diagnosis, suggesting that sex is a predictive factor, a finding corroborated by the present study. Chen et al. [55] further reported that lower parental educational attainment was associated with more severe and persistent sensory dysfunction in offspring. Collectively, these findings indicate that children’s sensory processing is influenced not only by biological and neurodevelopmental factors but also by environmental factors and sociodemographic characteristics, including sex, age, family structure, and parental education level. Although some associations appear statistically significant compared to others, their practical implications may differ in magnitude. Stronger effects observed in younger children may reflect the developmental maturation of sensory systems. Also, parental perceptions of children’s sensory behaviors may be influenced by cultural expectations and norms within the Greek context, highlighting the importance of culturally adapted assessment tools to ensure accurate interpretation.

Furthermore, given the absence of other valid and standardized instruments assessing sensory processing in the Greek population, and considering that SSP2 is among the most widely used and psychometrically robust instruments for evaluating sensory processing in children, the SCQ and the CBCL were selected to examine convergent validity in the present study. The moderate-to-strong correlations observed between SSP2 and CBCL scales suggest partial construct overlap, indicating that sensory processing difficulties are meaningfully associated with behavioral outcomes. However, the SSP2 primarily captures sensory processing patterns rather than behavioral disorders, underscoring its role as a screening rather than a diagnostic instrument.

Regarding the correlations between SSP2 and the additional instruments, statistically significant associations were observed between the Sensory scale and the SCQ (r = 0.311, *p* < 0.001), as well as between the Behavioral scale and the SCQ (r = 0.326, *p* < 0.001). These findings align with previous research demonstrating direct associations between sensory processing difficulties and communication and social challenges [56,57]. Moreover, statistically significant correlations emerged between the SSP2 and the CBCL (Supplementary Tables S1 and S2). Specifically, children classified within the clinical range of the CBCL scored higher on both the Sensory and Behavioral scales of the SSP2. This result is consistent with a large body of research indicating that sensory processing difficulties are strongly associated with emotional and behavioral problems, particularly during preschool and school-age years. More specifically, subscales reflecting elevated sensory seeking, sensitivity, or avoidance were found to be significantly associated with difficulties including anxiety, withdrawal, and both internalizing and externalizing problems [53,58,59,60].

Correlational analyses revealed that higher SSP2 scores were moderately associated with increased behavioral difficulties (CBCL) and autism-related traits (SCQ), consistent with prior evidence linking atypical sensory processing to behavioral and social-emotional challenges. While these correlations support convergent validity, it is important to emphasize that the SSP2 primarily assesses sensory processing patterns. Elevated SSP2 scores do not necessarily indicate broad behavioral or communicative problems, but rather reflect differences in sensory responsiveness. Clinicians should interpret elevated scores alongside other behavioral and developmental assessments, considering them as screening indicators that highlight areas where children may benefit from targeted support or individualized interventions. High SSP2 scores alone do not provide a standalone diagnosis but inform clinical reasoning and planning in the context of the child’s overall developmental profile. This guidance helps ensure that SSP2 results are used appropriately within both research and clinical practice. This interpretation aligns with the multifactorial nature of neurodevelopmental functioning and reinforces the utility of the SSP2 as a screening, rather than diagnostic, instrument. Using culturally adapted and psychometrically reliable assessment tools ensures that children with atypical sensory processing are appropriately identified for early support or intervention.

Several limitations of the present study should be acknowledged. Although subgroup differences by sex and age were explored descriptively, formal measurement invariance was not examined. Future research should investigate whether the factor structure and score interpretations of the Greek SSP2 remain stable across demographic groups. Confirmatory Factor Analysis (CFA) was not conducted in this study; therefore, subsequent research should include CFA in independent samples to verify the two-factor structure and assess invariance. In addition, in order to preserve participants’ anonymity, a test–retest reliability analysis could not be performed, limiting the evaluation of temporal stability. Finally, reliance on caregiver-report questionnaires introduces the possibility of response bias, as responses may be influenced by subjective perceptions or socially desirable answering tendencies. Although the findings provide meaningful insights for screening purposes, generalization to clinical populations should be approached cautiously as this study was conducted within a general population sample.

## 5. Conclusions

In conclusion, the Greek version of the SSP2 constitutes a valuable and psychometrically sound instrument for assessing sensory processing patterns and identifying their subtypes. The present study represents the first attempt to examine and validate the questionnaire’s psychometric properties in Greece, drawing on data from diverse geographical regions of the general population (Attica, mainland Greece, and islands). The findings provide a reliable foundation for researchers and healthcare professionals, including medical doctors and therapists, to evaluate sensory processing in children aged 3.0 to 14.11 years. The demonstrated reliability and construct validity of the Greek SSP2 support its use as a culturally adapted screening tool within both clinical and research settings. Following the validation of its psychometric properties in this age group, future research should extend the investigation of the Sensory Profile to other developmental stages, including infants, older adolescents, and adults, in order to examine developmental continuity and age-related differences in sensory processing. Despite certain methodological constraints, the study highlights the importance of sensory processing and its associated disorders as a domain warranting further systematic investigation. Future research may benefit from incorporating longitudinal designs to explore how sensory processing patterns evolve over time in Greek children and how these patterns relate to broader developmental trajectories and functional outcomes. Additionally, cross-informant comparisons (e.g., parent versus teacher or medical reports) and the integration of objective methods—such as structured observational protocols, physiological indices, biomarkers, neuroimaging, or genetic approaches—could provide a more comprehensive understanding of sensory processing and further strengthen the empirical foundation of the Greek version of the SSP2.

## Figures and Tables

**Table 1:** Sociodemographic Characteristics of the Sample (*n* = 350).

Sex of Parents *n* (%)	
Male	122 (34.9%)
Female	228 (65.1%)
Age of Parents	
Median (IQR)	39 (10.25)
Marital Status *n* (%)	
Married	299 (85.4%)
Other	51 (14.6%)
Nationality *n* (%)	
Greek	334 (95.4%)
Other	16 (4.6%)
Place of residence *n* (%)	
Attica	196 (56.0%)
Mainland Greece	102 (29.1%)
Islands	52 (14.9%)
Educational Level *n* (%)	
Until Upper Secondary School	47 (13.4%)
Post-Secondary/Tertiary	206 (58.9%)
MSc/PhD	97 (27.7%)
Work Status *n* (%)	
Employed	316 (90.3%)
Other	34 (9.7%)
Fathers’ Occupation *n* (%)	
Public Sector Employee	109 (31.1%)
Private Sector Employee	111 (31.7%)
Self-employed	121 (34.6%)
Other	9 (2.6%)
Mothers’ Occupation *n* (%)	
Public Sector Employee	116 (33.1%)
Private Sector Employee	128 (36.6%)
Self-employed	85 (24.3%)
Other	21 (6.0%)
Number of Children	
Median (IQR)	2 (1)
Sex of Child *n* (%)	
Male	193 (55.1%)
Female	157 (44.9%)
Age of Child	
Median (IQR)	6.11 (4.04)
Birth Order *n* (%)	
Only Child	98 (28.0%)
1st	106 (30.3%)
2nd	110 (31.4%)
Other	36 (10.3%)
More than 3 Children *n* (%)	
Yes	37 (10.6%)
No	313 (89.4%)
Completed by *n* (%)	
Father	122 (34.9%)
Mother	228 (65.1%)
Relation with the Child *n* (%)	
Biological Parent	348 (99.4%)
Other	2 (0.6%)
SCQ Score	
Median (IQR)	5.00 (6.00)

**Table 2:** Range, Mean (SD), Median (IQR), and Cronbach’s alpha coefficient for the 34 items of the SSP2 scales (*n* = 350).

Scale	Range	Mean (SD) Median (IQR)	Min–Max	Alpha of Scale
**Sensory**	70	22.09 (12.33) 19 (12)	0–70	0.939
**Behavioral**	100	34.25 (19.40) 29 (21)	0–100	0.968
**Seeking**	35	11.91 (6.65) 10 (8)	0–35	0.884
**Avoiding**	45	15.78 (9.07) 14 (9)	0–45	0.940
**Sensitivity**	50	17.85 (10.26) 16 (11)	0–50	0.942
**Registration**	40	10.65 (6.61) 9 (5)	0–40	0.910

**Table 4:** Correlations (Spearman’s rho) of quantitative variables with the SSP2 scales (*n* = 350).

	Sensory Correlation Coefficient *p*-Value	Behavioral Correlation Coefficient *p*-Value
**Sensory**		0.872 <0.001
**Behavioral**	0.872 <0.001	
**SCQ**	0.311 <0.001	0.326 <0.001
**Parental Age**	−0.328 <0.001	−0.340 <0.001
**Child’s Age**	−0.394 <0.001	−0.400 <0.001
**Number of Children**	−0.165 0.002	−0.178 0.001

**Table 5:** Cut-off points for the Greek standardization of the SSP2 (*n* = 350).

	Less Than Others	Just like the Majority of Others	More Than Others
**Seeking/Seeker**	0…………5	6…………18	19…………35
**Avoiding/Avoider**	0…………6	7…………25	26…………45
**Sensitivity/Sensor**	0…………7	8…………28	29…………50
**Registration/Bystander**	0…………4	5…………17	18…………40
**Sensory**	0…………9	10…………34	35…………70
**Behavioral**	0…………14	15…………53	54…………100

## Data Availability

The data supporting the findings of this study are not publicly available due to ethical and privacy restrictions involving minors. De-identified datasets may be provided by the corresponding author upon reasonable request and subject to approval by the institutional ethics committee.

## Supplementary Materials

The following supporting information can be downloaded at: https://www.mdpi.com/article/10.3390/children13030315/s1, Table S1: CBCL outcomes for the total sample (*n* = 350). Table S2: Association of the CBCL outcomes with the SSP2 scales (*n* = 350).

## References

[1] Dubois J., Dehaene-Lambertz G., Kulikova S., Poupon C., Hüppi P.S., Hertz-Pannier L. (2014). The early development of brain white matter: A review of imaging studies in fetuses, newborns and infants. Neuroscience.

[2] Greven C.U., Lionetti F., Booth C., Aron E.N., Fox E., Schendan H.E., Pluess M., Bruining H., Acevedo B., Bijttebier P. (2019). Sensory Processing Sensitivity in the context of Environmental Sensitivity: A critical review and development of research agenda. Neurosci. Biobehav. Rev..

[3] Dunn W. (2001). The sensations of everyday life: Empirical, theoretical, and pragmatic considerations. Am. J. Occup. Ther..

[4] Lane S.J., Mailloux Z., Schoen S., Bundy A., May-Benson T.A., Parham L.D., Smith Roley S., Schaaf R.C. (2019). Neural Foundations of Ayres Sensory Integration®. Brain Sci..

[5] Shahbazi M., Mirzakhani N. (2021). Assessment of Sensory Processing Characteristics in Children Between 0 and 14 Years of Age: A Systematic Review. Iran. J. Child Neurol..

[6] Gadhvi M., Moore M.J., Waseem M. (2023). Physiology, Sensory System. StatPearls.

[7] Dunn W. (1997). The impact of sensory processing abilities on the daily lives of young children and their families: A conceptual model. Infants Young Child..

[8] Park H.D., Blanke O. (2019). Coupling Inner and Outer Body for Self-Consciousness. Trends Cogn. Sci..

[9] Metz A.E., Boling D., DeVore A., Holladay H., Liao J.F., Vlutch K.V. (2019). Dunn’s Model of Sensory Processing: An Investigation of the Axes of the Four-Quadrant Model in Healthy Adults. Brain Sci..

[10] Ayres A.J., Robbins J., Pediatric Therapy Network (2005). Sensory Integration and the Child: Understanding Hidden Sensory Challenges.

[11] Celik H.I., Elbasan B., Gucuyener K., Kayihan H., Huri M. (2018). Investigation of the Relationship Between Sensory Processing and Motor Development in Preterm Infants. Am. J. Occup. Ther..

[12] Fischer K.W. (1980). A theory of cognitive development: The control and construction of hierarchies of skills. Psychol. Rev..

[13] Pavão S.L., Rocha N.A.C.F. (2017). Sensory processing disorders in children with cerebral palsy. Infant Behav. Dev..

[14] Greenspan S.I. (2004). Greenspan Social-Emotional Growth Chart: A Screening Questionnaire for Infants and Young Children.

[15] Brown A., Tse T., Fortune T. (2019). Defining sensory modulation: A review of the concept and a contemporary definition for application by occupational therapists. Scand. J. Occup. Ther..

[16] Fernandez-Prieto M., Moreira C., Cruz S., Campos V., Martínez-Regueiro R., Taboada M., Carracedo A., Sampaio A. (2021). Executive Functioning: A Mediator Between Sensory Processing and Behaviour in Autism Spectrum Disorder. J. Autism Dev. Disord..

[17] Passarello N., Tarantino V., Chirico A., Menghini D., Costanzo F., Sorrentino P., Fucà E., Gigliotta O., Alivernini F., Oliveri M. (2022). Sensory Processing Disorders in Children and Adolescents: Taking Stock of Assessment and Novel Therapeutic Tools. Brain Sci..

[18] Gouze K.R., Hopkins J., LeBailly S.A., Lavigne J.V. (2009). Re-examining the epidemiology of sensory regulation dysfunction and comorbid psychopathology. J. Abnorm. Child Psychol..

[19] Tomchek S.D., Dunn W. (2007). Sensory processing in children with and without autism: A comparative study using the short sensory profile. Am. J. Occup. Ther..

[20] McArthur A.L.-H. (2022). The debate over sensory processing disorder. Am. J. Psychiatry Resid. J..

[21] Hertzog D., Cermak S., Bar-Shalita T. (2019). Sensory modulation, physical activity and participation in daily occupations in young children. Can. J. Occup. Ther..

[22] Schaaf R.C., Toth-Cohen S., Johnson S.L., Outten G., Benevides T.W. (2011). The everyday routines of families of children with autism: Examining the impact of sensory processing difficulties on the family. Autism.

[23] Ismael N., Lawson L.M., Hartwell J. (2018). Relationship Between Sensory Processing and Participation in Daily Occupations for Children With Autism Spectrum Disorder: A Systematic Review of Studies That Used Dunn’s Sensory Processing Framework. Am. J. Occup. Ther..

[24] Nieto C., López B., Gandía H. (2017). Relationships between atypical sensory processing patterns, maladaptive behaviour and maternal stress in Spanish children with autism spectrum disorder. J. Intellect. Disabil. Res..

[25] Harris J. (2018). Leo Kanner and autism: A 75-year perspective. Int. Rev. Psychiatry.

[26] American Psychiatric Association, DSM-5 Task Force (2013). Diagnostic and Statistical Manual of Mental Disorders: DSM-5™.

[27] Cheung P.P.P., Lau B.W.M. (2020). Neurobiology of sensory processing in autism spectrum disorder. Prog. Mol. Biol. Transl. Sci..

[28] Lane A.E., Young R.L., Baker A.E., Angley M.T. (2010). Sensory processing subtypes in autism: Association with adaptive behavior. J. Autism Dev. Disord..

[29] Schaaf R.C., Dumont R.L., Arbesman M., May-Benson T.A. (2018). Efficacy of Occupational Therapy Using Ayres Sensory Integration®: A Systematic Review. Am. J. Occup. Ther..

[30] Little L.M., Dean E., Tomchek S., Dunn W. (2018). Sensory Processing Patterns in Autism, Attention Deficit Hyperactivity Disorder, and Typical Development. Phys. Occup. Ther. Pediatr..

[31] Pfeiffer B., Daly B.P., Nicholls E.G., Gullo D.F. (2015). Assessing sensory processing problems in children with and without attention deficit hyperactivity disorder. Phys. Occup. Ther. Pediatr..

[32] Hassan D.M., Azzam H., Norvilitis J.M. (2012). Sensory integration in attention deficit hyperactivity disorder: Implications to postural control. Contemporary Trends in ADHD Research.

[33] Anquetil M., Roche-Labarbe N., Rossi S. (2023). Tactile sensory processing as a precursor of executive attention: Toward early detection of attention impairments and neurodevelopmental disorders. Wiley Interdiscip. Rev. Cogn. Sci..

[34] Román-Oyola R., Reynolds S. (2013). Prevalence of sensory modulation disorder among Puerto Rican preschoolers: An analysis focused on socioeconomic status variables. Occup. Ther. Int..

[35] Delgado-Lobete L., Pértega-Díaz S., Santos-Del-Riego S., Montes-Montes R. (2020). Sensory processing patterns in developmental coordination disorder, attention deficit hyperactivity disorder and typical development. Res. Dev. Disabil..

[36] Dunn W. (1999). Sensory Profile.

[37] World Health Organization (2022). Process of Translation and Adaptation of Instruments.

[38] Jorquera-Cabrera S., Romero-Ayuso D., Rodriguez-Gil G., Triviño-Juárez J.M. (2017). Assessment of Sensory Processing Characteristics in Children between 3 and 11 Years Old: A Systematic Review. Front. Pediatr..

[39] Dunn W. (2014). Sensory Profile 2: User’s Manual.

[40] Achenbach T.M., Rescorla L.A. (2001). Manual for the ASEBA School-Age Forms and Profiles.

[41] Warnick E.M., Bracken M.B., Kasl S. (2008). Screening Efficiency of the Child Behavior Checklist and Strengths and Difficulties Questionnaire: A Systematic Review. Child Adolesc. Ment. Health.

[42] Roussos A., Karantanos G., Richardson C., Hartman C., Karajiannis D., Kyprianos S., Lazaratou H., Mahaira O., Tassi M., Zoubou V. (1999). Achenbach’s Child Behavior Checklist and Teachers’ Report Form in a normative sample of Greek children 6–12 years old. Eur. Child Adolesc. Psychiatry.

[43] Rutter M., Bailey A., Lord C. (2003). The Social Communication Questionnaire Manual.

[44] Karaminis T., Stavrakaki S. (2022). The psychometric properties of the Greek version of the Social Communication Questionnaire. Autism Res..

[45] IBM Corp (2019). *IBM SPSS Statistics for Windows*, Version 26.0.

[46] Dean E.E., Little L., Tomchek S., Wallisch A., Dunn W. (2022). Prevalence Models to Support Participation: Sensory Patterns as a Feature of All Children’s Humanity. Front. Psychol..

[47] Chojnicka I., Pisula E. (2019). Adaptation and psychometric properties of the Polish version of the Short Sensory Profile 2. Medicine.

[48] Im S.E., Loh S.Y., Chinna K., Marret M. (2016). Cross-cultural adaptation and psychometric properties of the Malay version of the Short Sensory Profile. Phys. Occup. Ther. Pediatr..

[49] Robertson C.E., Baron-Cohen S. (2017). Sensory perception in autism. Nat. Rev. Neurosci..

[50] Gigliotti F., Martelli M.E., Giovannone F., Sogos C. (2025). Feeling the World Differently: Sensory and Emotional Profiles in Preschool Neurodevelopmental Disorders. Children.

[51] Chen Y., Xi Z., Saunders R., Simmons D., Totsika V., Mandy W. (2024). A systematic review and meta-analysis of the relationship between sensory processing differences and internalising/externalising problems in autism. Clin. Psychol. Rev..

[52] Dellapiazza F., Michelon C., Oreve M.J., Robel L., Schoenberger M., Chatel C., Vesperini S., Maffre T., Schmidt R., Blanc N. (2020). The Impact of Atypical Sensory Processing on Adaptive Functioning and Maladaptive Behaviors in Autism Spectrum Disorder During Childhood: Results From the ELENA Cohort. J. Autism Dev. Disord..

[53] Gigliotti F., Giovannone F., Belli A., Sogos C. (2024). Atypical Sensory Processing in Neurodevelopmental Disorders: Clinical Phenotypes in Preschool-Aged Children. Children.

[54] Jussila K., Junttila M., Kielinen M., Ebeling H., Joskitt L., Moilanen I., Mattila M.L. (2020). Sensory Abnormality and Quantitative Autism Traits in Children With and Without Autism Spectrum Disorder in an Epidemiological Population. J. Autism Dev. Disord..

[55] Chen Y.J., Sideris J., Watson L.R., Crais E.R., Baranek G.T. (2024). Early developmental profiles of sensory features and links to school-age adaptive and maladaptive outcomes: A birth cohort investigation. Dev. Psychopathol..

[56] Khaledi H., Aghaz A., Mohammadi A., Mohammadi M. (2022). The relationship between communication skills, sensory difficulties, and anxiety in children with autism spectrum disorder. Middle East Curr. Psychiatry.

[57] Kojovic N., Ben Hadid L., Franchini M., Schaer M. (2019). Sensory Processing Issues and Their Association with Social Difficulties in Children with Autism Spectrum Disorders. J. Clin. Med..

[58] Fabbri-Destro M., Maugeri F., Ianni C., Corsini S., Di Stefano E., Scatigna S., Crifaci G., Bruzzi G., Berloffa S., Fantozzi P. (2022). Early Sensory Profile in Autism Spectrum Disorders Predicts Emotional and Behavioral Issues. J. Pers. Med..

[59] Sakamoto N., Oe M., Ozone M. (2024). An Exploratory Study of the Associations Between Sensory Processing Patterns and Emotional/Behavioral Problems in Children with Autism Spectrum Disorder. Kurume Med. J..

[60] Valencia M., D’Ocon A., Plata R., Simó S., Cantero M.J. (2025). Internalizing/Externalizing Problems and Sensory Processing Alteration in Children Referred to Child Mental Health Centers. Children.

