# Supplementary material for: Adaptation and Psychometric Validation of the Greek Version of the Sensory Profile 2 Short Form
Source: Children (Basel). 2026 Feb 24;13(3):315. doi: 10.3390/children13030315 (PMC13025351; doi:10.3390/children13030315)
Supplement: Supplementary file 1 [file children-13-00315-s001.zip › children-4157260-supplementary.pdf]

Supplementary Materials

Table S1 presents a detailed overview of the CBCL questionnaire results for the total sample.

Table S1. CBCL outcomes for the total sample (*n* = 350).

# **Activities** *n* (%)		• **Total Problems** *n* (%)	
Normal	147 (71.7%)	Normal	281 (80.3%)
Borderline Clinical	28 (13.7%)	Borderline Clinical	27 (7.7%)
Clinical	30 (14.6%)	Clinical	42 (12.0%)
# **Social** *n* (%)		• **Affective Problems** *n* (%)	
Normal	176 (85.9%)	Normal	306 (87.4%)
Borderline Clinical	21 (10.2%)	Borderline Clinical	20 (5.7%)
Clinical	8 (3.9%)	Clinical	24 (6.9%)
# **School** *n* (%)		• **Anxiety Problems** *n* (%)	
Normal	193 (94.1%)	Normal	285 (81.4%)
Borderline Clinical	6 (2.9%)	Borderline Clinical	37 (10.6%)
Clinical	6 (2.9%)	Clinical	28 (8.0%)
# **Total Competence** *n* (%)		# **Somatic Problems** *n* (%)	
Normal	121 (59.0%)	Normal	191 (93.2%)
Borderline Clinical	36 (17.6%)	Borderline Clinical	9 (4.4%)
Clinical	48 (23.4%)	Clinical	5 (2.4%)
• **Anxious/Depressed** *n* (%)		• **ADHD Problems** *n* (%)	
Normal	310 (88.6%)	Normal	275 (78.6%)
Borderline Clinical	23 (6.6%)	Borderline Clinical	44 (12.6%)
Clinical	17 (4.9%)	Clinical	31 (8.9%)
# **Withdrawn/Depressed** *n* (%)		• **Oppositional Defiant Problems** *n* (%)	
Normal	180 (87.8%)	Normal	324 (92.6%)
Borderline Clinical	16 (7.8%)	Borderline Clinical	18 (5.1%)
Clinical	9 (4.4%)	Clinical	8 (2.3%)
• **Somatic Complaints** *n* (%)		# **Conduct Problems** *n* (%)	
Normal	332 (94.9%)	Normal	189 (92.2%)
Borderline Clinical	8 (2.3%)	Borderline Clinical	9 (4.4%)
Clinical	10 (2.9%)	Clinical	7 (3.4%)
# **Social Problems** *n* (%)		# **Sluggish Cognitive Problems** *n* (%)	
Normal	183 (89.3%)	Normal	182 (88.8%)
Borderline Clinical	11 (5.4%)	Borderline Clinical	15 (7.3%)
Clinical	11 (5.4%)	Clinical	8 (3.9%)
# **Thought Problems** *n* (%)		# **Obsessive–Compulsive Problems** *n* (%)	
Normal	189 (92.2%)	Normal	184 (89.8%)
Borderline Clinical	7 (3.4%)	Borderline Clinical	9 (4.4%)
Clinical	9 (4.4%)	Clinical	12 (5.9%)
• **Attention Problems** *n* (%)		# **Post–traumatic Stress Problems** *n* (%)	
Normal	271 (77.4%)	Normal	182 (89.2%)
Borderline Clinical	39 (11.1%)	Borderline Clinical	10 (4.9%)
Clinical	40 (11.4%)	Clinical	12 (5.9%)
# **Rule–Breaking Behavior** *n* (%)		* **Emotionally Reactive** *n* (%)	
Normal	189 (92.2%)	Normal	106 (73.1%)
Borderline Clinical	9 (4.4%)	Borderline Clinical	17 (11.7%)
Clinical	7 (3.4%)	Clinical	22 (15.2%)
• **Aggressive Behavior** *n* (%)		* **Withdrawn** *n* (%)	
Normal	316 (90.3%)	Normal	111 (76.6%)
Borderline Clinical	22 (6.3%)	Borderline Clinical	14 (9.7%)
Clinical	12 (3.4%)	Clinical	20 (13.8%)

• **Internalizing Problems** *n* (%)		* **Sleep Problems** *n* (%)	
Normal	268 (76.6%)	Normal	140 (96.6%)
Borderline Clinical	26 (7.4%)	Borderline Clinical	0 (0%)
Clinical	56 (16.0%)	Clinical	5 (3.4%)
• **Externalizing Problems** *n* (%)		* **Pervasive Developmental Problems** *n* (%)	
Normal	284 (81.1%)	Normal	105 (72.4%)
Borderline Clinical	28 (8.0%)	Borderline Clinical	15 (10.3%)
Clinical	38 (10.9%)	Clinical	25 (17.2%)

Note: Results marked with * refer to children aged 3.0 to <6 years, results marked with # refer to children aged 6.0 to <15 years, and results marked with • refer to the total sample.

Table S2 presents the associations between CBCL outcomes and the SSP2 scales (Sensory and Behavioral) for the total sample.

Table S2. Association of the CBCL outcomes with the SSP2 scales (*n* = 350).

Study measurements	Categories	Sensory scale	Behavioral scale
# **Activities** Median (IQR)	Normal	17 (18.5)	25 (12)
	Borderline Clinical	17 (11.75)	22 (17.25)
	Clinical	17 (4.25)	25 (14.5)
	p-value	0.713	0.600
# **Social** Median (IQR)	Normal	17 (6)	23.5 (12.75)
	Borderline Clinical	18 (23.5)	36 (38)
	Clinical	34.5 (26.25)	60.5 (46.75)
	p-value	0.001	<0.001
# **School** Median (IQR)	Normal	17 (7)	24 (12.5)
	Borderline Clinical	33.5 (21)	63 (21.25)
	Clinical	41.5 (16.25)	70.5 (19.5)
	p-value	<0.001	<0.001
# **Total Competence** Median (IQR)	Normal	16 (9.5)	23 (13)
	Borderline Clinical	17 (8)	26.5 (13.5)
	Clinical	18 (15.75)	25.5 (22.75)
	p-value	0.017	0.006
• **Anxious/Depressed** Median (IQR)	Normal	18 (9.5)	27 (15)
	Borderline Clinical	35 (19)	60 (23)
	Clinical	40 (17.5)	69 (24.5)
	p-value	<0.001	<0.001
# **Withdrawn/Depressed** Median (IQR)	Normal	17 (5.75)	23 (11.75)
	Borderline Clinical	24.5 (14.25)	43.5 (28.75)
	Clinical	40 (15.5)	69 (16)
	p-value	<0.001	<0.001
• **Somatic Complaints** Median (IQR)	Normal	19 (12)	28 (19)
	Borderline Clinical	21.5 (25.75)	38.5 (45.75)
	Clinical	41.5 (16.75)	67.5 (24.5)
	p-value	<0.001	<0.001
# **Social Problems** Median (IQR)	Normal	16 (7)	23 (12)
	Borderline Clinical	29 (17)	56 (28)
	Clinical	35 (15)	64 (17)
	p-value	<0.001	<0.001
# **Thought Problems** Median (IQR)	Normal	17 (6.5)	24 (12)
	Borderline Clinical	16 (27)	38 (37)
	Clinical	40 (19.5)	69 (19)

	p-value	<0.001	<0.001
• **Attention Problems** Median (IQR)	Normal	17 (8)	25 (13)
	Borderline Clinical	30 (10)	49 (16)
	Clinical	47 (20.75)	72 (23.25)
	p-value	<0.001	<0.001
# **Rule–Breaking Behavior** Median (IQR)	Normal	17 (6.5)	24 (13)
	Borderline Clinical	25 (16)	55 (29)
	Clinical	38 (12)	69 (18)
	p-value	<0.001	<0.001
• **Aggressive Behavior** Median (IQR)	Normal	18 (10.75)	27 (15.75)
	Borderline Clinical	48 (24.25)	67.5 (25.75)
	Clinical	34.5 (14.25)	72.5 (23.25)
	p-value	<0.001	<0.001
• **Internalizing Problems** Median (IQR)	Normal	17 (8)	26 (13)
	Borderline Clinical	27 (16.5)	41.5 (24.5)
	Clinical	40 (19.5)	68.5 (29)
	p-value	<0.001	<0.001
• **Externalizing Problems** Median (IQR)	Normal	17 (8.75)	26 (13.75)
	Borderline Clinical	32 (23)	56 (26.5)
	Clinical	41.5 (22.5)	70.5 (24.25)
	p-value	<0.001	<0.001
• **Total Problems** Median (IQR)	Normal	17 (8.5)	26 (13.5)
	Borderline Clinical	30 (19)	57 (30)
	Clinical	45 (18.75)	72 (21)
	p-value	<0.001	<0.001
• **Affective Problems** Median (IQR)	Normal	18 (10)	27 (14.25)
	Borderline Clinical	33.5 (19)	57 (37)
	Clinical	51 (17.75)	81 (20.75)
	p-value	<0.001	<0.001
• **Anxiety Problems** Median (IQR)	Normal	17 (9)	26 (13.5)
	Borderline Clinical	28 (20)	43 (39)
	Clinical	42 (16.75)	71 (20)
	p-value	<0.001	<0.001
# **Somatic Problems** Median (IQR)	Normal	17 (7)	24 (12)
	Borderline Clinical	15 (16.5)	25 (21.5)
	Clinical	43 (12.5)	67 (10.5)
	p-value	0.001	0.002
• **ADHD Problems** Median (IQR)	Normal	17 (8)	25 (13)
	Borderline Clinical	35.5 (20)	56 (24.5)
	Clinical	41 (18)	70 (21)
	p-value	<0.001	<0.001
• **Oppositional Defiant Problems** Median (IQR)	Normal	18 (10)	28 (17.75)
	Borderline Clinical	28.5 (22.5)	64 (27.25)
	Clinical	38 (17.25)	79 (20.75)
	p-value	<0.001	<0.001
# **Conduct Problems** Median (IQR)	Normal	17 (6.5)	24 (13)
	Borderline Clinical	25 (17)	45 (40)
	Clinical	38 (12)	69 (18)
	p-value	<0.001	<0.001
# **Sluggish Cognitive Problems** Median (IQR)	Normal	16.5 (6.25)	23 (12)
	Borderline Clinical	32 (12)	55 (25)

	Clinical	38 (14.25)	71.5 (23)
	p-value	<0.001	<0.001
# **Obsessive–Compulsive Problems** Median (IQR)	Normal	17 (7)	23 (12)
	Borderline Clinical	16 (10.5)	35 (13)
	Clinical	38 (21.25)	68 (15.25)
	p-value	<0.001	<0.001
# **Post–traumatic Stress Problems** Median (IQR)	Normal	17 (5.5)	23 (11.25)
	Borderline Clinical	23.5 (20.75)	51.5 (29.5)
	Clinical	38 (14.5)	69 (20.25)
	p-value	<0.001	<0.001
* **Emotionally Reactive** Median (IQR)	Normal	22 (11)	33 (15.25)
	Borderline Clinical	32 (16)	57 (24)
	Clinical	51 (19)	79 (17.25)
	p-value	<0.001	<0.001
* **Withdrawn** Median (IQR)	Normal	22 (11)	33 (16)
	Borderline Clinical	31 (23.5)	58 (31)
	Clinical	50 (17.75)	77.5 (17.75)
	p-value	<0.001	<0.001
* **Sleep Problems** Median (IQR)	Normal	24 (17.75)	34 (28.75)
	Borderline Clinical	-	-
	Clinical	31 (46)	80 (67)
	p-value	0.362	0.113
* **Pervasive Developmental Problems** Median (IQR)	Normal	22 (9)	33 (13.5)
	Borderline Clinical	33 (20)	49 (29)
	Clinical	51 (19.5)	77 (21.5)
	p-value	<0.001	<0.001

Kruskal Wallis test, $p < 0.05$. Note: Results marked with * refer to children aged 3.0 to <6 years, results marked with # refer to children aged 6.0 to <15 years, and results marked with • refer to the total sample.
